# Short-Term Efficacy of Early Sodium–Glucose Cotransporter 2 Inhibitor Initiation in Patients With Heart Failure After Acute Anterior Myocardial Infarction

**DOI:** 10.31083/RCM48719

**Published:** 2026-08-19

**Authors:** Shuli Bi, Dong Li, Jianping Du, Wenting Li, Xiaoyu Yang, Wenqi Tao, Yan Zhang, Weiyuan Zhang, Zhuhua Yao

**Affiliations:** ^1^School of Medicine, Nankai University, 300071 Tianjin, China; ^2^Department of Cardiology, Tianjin Union Medical Center, The First Affiliated Hospital of Nankai University, 300121 Tianjin, China; ^3^The Institute of Translational Medicine, Tianjin Union Medical Center of Nankai University, 300121 Tianjin, China

**Keywords:** anterior wall myocardial infarction, heart failure, sodium–glucose cotransporter 2 inhibitors

## Abstract

**Background:**

Patients with heart failure (HF) following acute anterior ST-segment elevation myocardial infarction (STEMI) are at high risk for adverse ventricular remodeling, and a significant residual risk persists despite optimal guideline-directed medical therapy (GDMT). Although sodium–glucose cotransporter 2 (SGLT2) inhibitors have demonstrated clear benefit in chronic HF, evidence is limited regarding the early initiation of these inhibitors after acute myocardial infarction (MI), particularly in patients with HF complicating anterior wall infarction. Therefore, this study aimed to evaluate the short-term effects of early initiation of the SGLT2 inhibitor (SGLT2i) empagliflozin, when added to standard medical therapy, on cardiac function and safety in patients with HF following acute anterior STEMI.

**Methods:**

This single-center retrospective cohort study consecutively enrolled 98 patients hospitalized at Tianjin Union Medical Center between January 2022 and December 2022 with acute anterior STEMI complicated by HF. Patients were divided into two groups based on SGLT2i use: the control group (n = 49) received standard medical therapy alone, while the SGLT2i group (n = 49) received empagliflozin 10 mg once daily in addition to standard therapy. The primary outcome was the change in left ventricular ejection fraction (LVEF) from admission to one month after discharge. Analysis of covariance (ANCOVA) and multivariable linear regression models were employed to adjust for potential confounders.

**Results:**

Baseline characteristics were well-balanced between the two groups. At one-month follow-up, the SGLT2i group showed a significantly greater improvement in LVEF than the control group, with a multivariable-adjusted effect size of 1.92% (95% confidence interval (CI): 0.44–3.40%; *p* = 0.012). Serum uric acid levels were significantly lower in the SGLT2i group than in the control group (*p* = 0.006). In both groups, N-terminal pro-brain natriuretic peptide (NT-proBNP) levels decreased from baseline to one-month follow-up, with no significant between-group difference. The incidence of adverse events was similar between groups, and no cases of ketoacidosis or severe deterioration in renal function were observed.

**Conclusion:**

In hemodynamically stable patients with acute anterior STEMI complicated by HF, early initiation of SGLT2 inhibitors was associated with higher LVEF at 1 month and a trend toward lower uric acid levels, without significant differences in safety outcomes between groups. These preliminary findings suggest potential benefit and warrant confirmation in prospective studies.

## 1. Introduction

Acute myocardial infarction (AMI) is a leading cause of morbidity and mortality worldwide. Although advances in reperfusion therapy and antiplatelet regimens have substantially reduced short-term mortality following AMI, the incidence of post-infarction heart failure (HF) remains persistently high, with approximately one-third of patients progressing to chronic HF after the index event [1]. The development of HF after AMI is strongly associated with increased risks of hospital readmission and mortality, while presenting a substantial healthcare burden. Ventricular remodeling is defined as progressive changes in left ventricular size, morphology, and function following myocardial infarction (MI). It is widely recognized as a key pathological mechanism underlying post-infarction HF [2]. Current guideline-directed medical therapy (GDMT), including angiotensin-converting enzyme inhibitors/angiotensin receptor blockers, beta-blockers, and mineralocorticoid receptor antagonists, has been demonstrated to effectively attenuate adverse ventricular remodeling [3]. However, despite optimal GDMT, a significant residual risk of post-infarction HF persists, underscoring the pressing need for novel therapeutic strategies in this population.

Sodium-glucose cotransporter 2 (SGLT2) inhibitors, initially developed as glucose-lowering agents, have emerged as a cornerstone treatment in cardiovascular medicine. In recent years, multiple randomized controlled trials have established that SGLT2 inhibitors (SGLT2i) significantly improve cardiovascular outcomes in patients with type 2 diabetes mellitus [4], chronic kidney disease [5], and chronic HF irrespective of ejection fraction [6,7]. Notably, SGLT2i represent the only drug class proven to confer benefit across the entire spectrum of left ventricular ejection fraction (LVEF) in HF [8]. However, evidence regarding their role in the post-AMI setting remains inconclusive. Two recently published, large-scale randomized controlled trials—DAPA-MI [9] and EMPACT-MI [10]—evaluated the efficacy of dapagliflozin and empagliflozin, respectively, in patients following AMI. Although both trials yielded neutral results for their primary endpoints (cardiovascular death or hospitalization for HF), exploratory analyses suggested that SGLT2i may reduce the risk of hospitalization for HF (EMPACT-MI) and improve metabolic outcomes (DAPA-MI). These findings imply that SGLT2i could still hold promise in the early management of post-AMI patients, particularly among high-risk subgroups.

Anterior wall MI involves a larger myocardial territory and is associated with a 1.9–fold higher risk of adverse ventricular remodeling compared with infarcts in other locations (e.g., inferior or posterior walls) [11], rendering these patients particularly vulnerable to post-infarction HF. Nevertheless, data specifically addressing the early initiation of SGLT2i in patients with acute anterior wall MI complicated by HF remain limited. The aim of this real-world study was therefore to evaluate the short-term effects of early SGLT2i initiation, when added to standard medical therapy, on cardiac function (LVEF) and safety in patients with acute anterior ST-segment elevation myocardial infarction (STEMI) complicated by HF.

## 2. Methods

### 2.1 Study Design

This single-center retrospective cohort study consecutively enrolled patients admitted to the Coronary Care Unit (CCU) of Tianjin Union Medical Center between January 2022 and December 2022 with a diagnosis of acute anterior STEMI complicated by HF. All patients received background pharmacological therapy, including antiplatelet agents, loop diuretics, beta-blockers, mineralocorticoid receptor antagonists (MRA), and initiating doses of an angiotensin receptor-neprilysin inhibitor (ARNI), angiotensin-converting enzyme inhibitor (ACEI), or angiotensin II receptor blocker (ARB). Patients were divided into two groups based on the use of SGLT2 inhibitor: the control group (n = 49) received standard medical therapy alone, while the SGLT2i group (n = 49) received empagliflozin 10 mg orally once daily in addition to standard therapy. The study protocol was approved by the Ethics Committee of Tianjin Union Medical Center (IRB number: 2026-B177). Given the retrospective observational nature of the study, the absence of additional interventions, and the anonymization of all patient data to ensure confidentiality, the requirement for informed consent was waived. The study was conducted in accordance with the Declaration of Helsinki.

### 2.2 Study Population

**Fig. 1.**
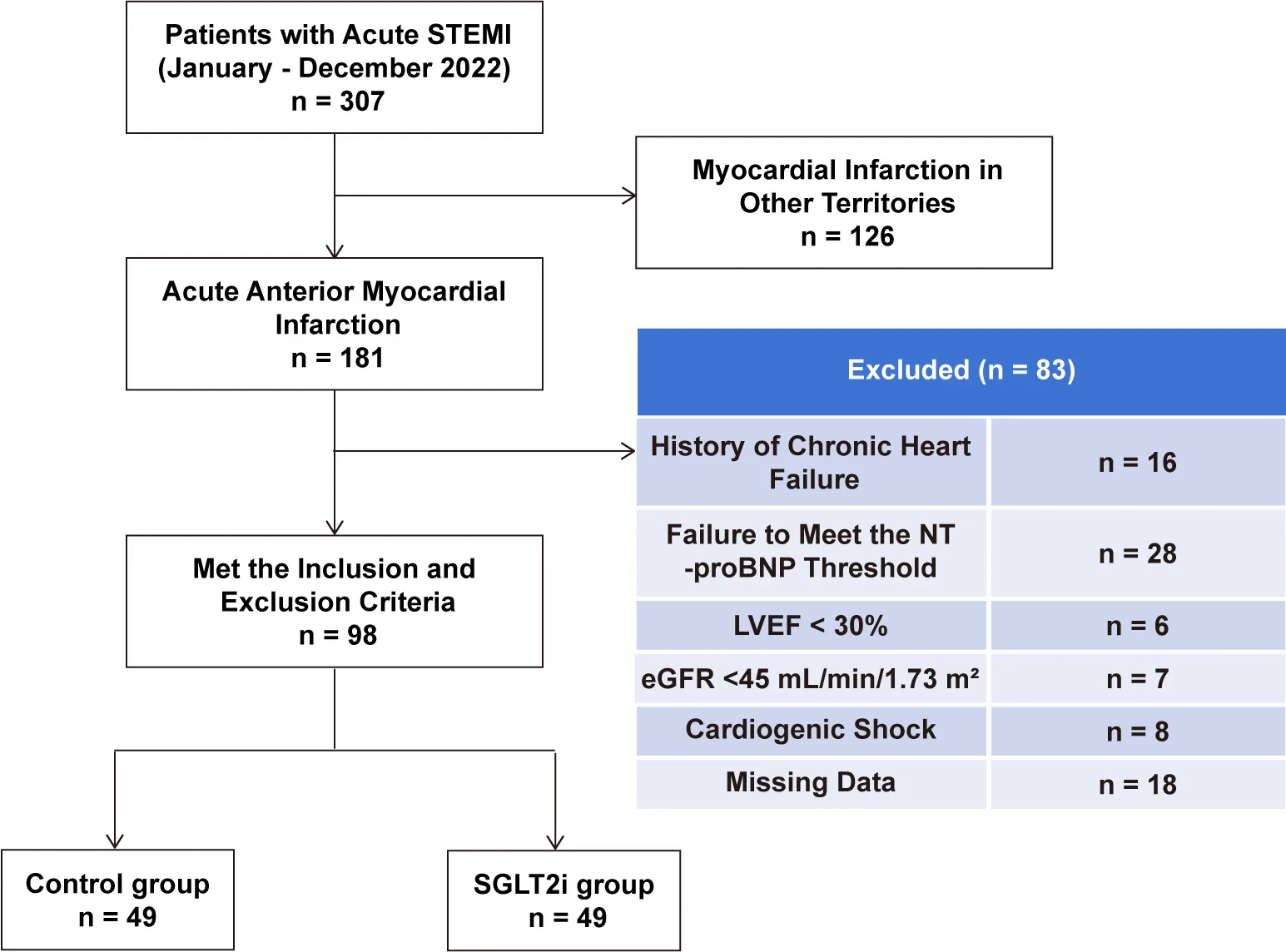
**Flowchart of patient selection**. STEMI, ST-segment elevation myocardial infarction; SGLT2i, SGLT2 inhibitor; NT-proBNP, N-terminal pro-B-type natriuretic peptide; LVEF, left ventricular ejection fraction; eGFR, estimated glomerular filtration rate.

The patient selection process is outlined in Fig. 1. Patients were included if they met the following criteria: (1) age ≥18 years; (2) diagnosis of Type 1 MI according to the Fourth Universal Definition of Myocardial Infarction [12], with definitive evidence of acute anterior STEMI; (3) diagnosis of acute post-infarction HF based on the 2020 Chinese Expert Consensus on the Prevention and Treatment of Heart Failure After Myocardial Infarction (Table 1) [13]; (4) N-terminal pro-brain natriuretic peptide (NT-proBNP) level ≥300 pg/mL following the index MI (≥900 pg/mL for patients with atrial fibrillation) [14]; and (5) hemodynamic stability upon admission, defined as systolic blood pressure ≥95 mmHg. Exclusion criteria were: (1) history of chronic HF; (2) development of cardiogenic shock during the index hospitalization for MI; (3) LVEF <30%; and (4) estimated glomerular filtration rate (eGFR) <45 mL/min/1.73 m^2^.

**Table 1.** **Diagnostic criteria for HF following myocardial infarction classified by LVEF [13]**.

Criteria	HFrEF	HFmrEF	HFpEF
1	History of Myocardial Infarction	History of Myocardial Infarction	History of Myocardial Infarction
2	Symptoms and/or Signs of Heart Failure	Symptoms and/or Signs of Heart Failure	Symptoms and/or Signs of Heart Failure
3	LVEF <40%	LVEF 40%–49%	LVEF ≥50%
4	Other causes of heart failure excluded	Elevated natriuretic peptides* AND at least one of the following criteria met: (1) Left ventricular hypertrophy and/or left atrial enlargement; (2) Evidence of abnormal diastolic dysfunction	Elevated natriuretic peptides* AND at least one of the following criteria met: (1) Left ventricular hypertrophy and/or left atrial enlargement; (2) Evidence of abnormal diastolic dysfunction
5	–	Other causes of heart failure excluded	Other causes of heart failure excluded

Note: HFrEF, heart failure with reduced ejection fraction; HFmrEF, heart failure with mildly reduced ejection fraction; HFpEF, heart failure with preserved ejection fraction; LVEF, left ventricular ejection fraction. *: Elevated natriuretic peptides defined as B-type natriuretic peptide >35 ng/L and/or N-terminal pro-B-type natriuretic peptide >125 ng/L. –: Not applicable.

### 2.3 Definitions

#### 2.3.1 Myocardial Infarction Type 1

MI caused by atherothrombotic coronary artery disease (CAD) and usually precipitated by atherosclerotic plaque disruption (rupture or erosion) was designated as a type 1 MI [12].

#### 2.3.2 Criteria for Type 1 MI

Detection of a rise and/or fall of cardiac troponin (cTn) values, with at least one value above the 99th percentile URL and with at least one of the following: (1) symptoms of acute myocardial ischemia; (2) new ischemic electrocardiogram (ECG) changes; (3) development of pathological Q waves; (4) imaging evidence of new loss of viable myocardium or new regional wall motion abnormality in a pattern consistent with an ischemic etiology; (5) identification of a coronary thrombus by angiography including intracoronary imaging or by autopsy [12].

Postmortem demonstration of an atherothrombus in the artery supplying the infarcted myocardium, or a macroscopically large circumscribed area of necrosis with or without intramyocardial hemorrhage, meets the type 1 MI criteria regardless of cTn values.

#### 2.3.3 eGFR

The eGFR was uniformly calculated using the Cockcroft-Gault formula: For male patients, eGFR = [(140 – age) × weight (kg)] / [0.818 × serum creatinine (μmol/L)]; for female patients, the result was multiplied by 0.85. The unit of measurement is mL/min/1.73 m^2^.

### 2.4 Treatment and Group Allocation

Patients in the SGLT2i group received empagliflozin (10 mg orally once daily) in addition to standard medical therapy. This was initiated early after admission, with a median time of 2 days (interquartile range [IQR]: 2–3 days) post-admission. Prior to treatment initiation, all patients met the predefined criteria for hemodynamic stability (systolic blood pressure ≥95 mmHg; median systolic blood pressure 132.0 mmHg, IQR: 120–149 mmHg) and renal function threshold (eGFR ≥45 mL/min/1.73 m^2^; median eGFR 124.9 mL/min/1.73 m^2^, IQR: 99–147 mL/min/1.73 m^2^). Treatment adherence was defined as the continued possession of an empagliflozin prescription with documented consistent use at one-month follow-up, based on medical records.

### 2.5 Echocardiographic Assessment

All patients underwent transthoracic echocardiography within 48 h of admission (prior to SGLT2i initiation) and at 30 ± 3 days after discharge. Raw images were retrieved from the hospital’s Picture Archiving and Communication System (PACS), anonymized, and independently re-analyzed by two experienced cardiac sonographers, each with >5 years of experience. Both sonographers were blinded to treatment group allocation and image timing (baseline vs. follow-up).

LVEF was measured strictly in accordance with the American Society of Echocardiography guidelines [15], primarily using the biplane Simpson’s method. For cases with suboptimal image quality precluding Simpson’s method analysis (3% of studies), LVEF was determined by visual estimation through consensus between the two sonographers. Three-dimensional echocardiography was not employed in this study.

To quantify measurement reliability, a random sample of 20 studies (including both baseline and follow-up images) was selected for test-retest analysis. Intra-observer variability was assessed by having the same sonographer repeat measurements two weeks after the initial analysis, and blinded to the previous results. Inter-observer variability was evaluated by comparing measurements between the two independent sonographers. Reliability was assessed using the intraclass correlation coefficient (ICC) based on a two-way random-effects model with absolute agreement. The analysis demonstrated excellent reproducibility, with an intra-observer ICC of 0.95 (95% confidence interval [CI]: 0.91–0.98) and an inter-observer ICC of 0.92 (95% CI: 0.88–0.95). The mean absolute difference between measurements was 2.8% ± 1.5%, confirming low measurement variability in this study.

### 2.6 Data Collection

Patient data were collected from the electronic medical record system. These included: (1) demographic characteristics, including age, sex, heart rate (HR), blood pressure (BP), and smoking status; (2) clinical background, including history of hypertension, diabetes mellitus (DM), atrial fibrillation, ischemic stroke, old myocardial infarction (OMI), peripheral vascular disease (PVD), dyslipidemia, and chronic kidney disease (CKD); (3) laboratory parameters, including NT-proBNP, peak creatine kinase (CK), blood glucose, glycated hemoglobin (HbA1c), lipid profile, liver function tests, renal function tests, and electrolytes; (4) echocardiographic parameters, including LVEF; (5) medication use, including diuretics, beta-blockers, ARNI/ACEI/ARB, MRAs, lipid-lowering agents, glucose-lowering drugs, and antiplatelet agents; and (6) safety outcomes, including hypotension, hyperkalemia, severe renal function deterioration, urinary tract infection, ketoacidosis, and heart failure hospitalization.

### 2.7 Study Outcomes

The primary outcome measure was the change in LVEF from admission to one month after discharge.

Exploratory outcomes included: (1) NT-proBNP levels, including the peak value within 72 h of admission, and the level at one-month post-treatment; and (2) the effects of treatment on blood glucose, lipid profile, liver function, renal function, and electrolyte levels.

Safety outcomes were the incidence of adverse events, including hypoglycemia, hypotension, urinary tract infection, genital infection, diabetic ketoacidosis, and amputation.

### 2.8 Statistical Analysis

Statistical analyses were performed using IBM SPSS Statistics version 25.0 (IBM Corp., Armonk, NY, USA). Continuous variables with a normal distribution were presented as the mean ± standard deviation (SD), with intergroup comparisons conducted using the independent samples *t*-test, and intragroup comparisons using the paired samples *t*-test. Continuous variables with a non-normal distribution were presented as the median (interquartile range), with intergroup comparisons performed using the Mann-Whitney U test, and intragroup comparisons using the Wilcoxon signed-rank test. Categorical variables were presented as frequencies and percentages (%), with intergroup comparisons performed using the Chi-square test, or Fisher’s exact test when expected frequencies were <5.

Linear regression typically requires a minimum of 10 observations per predictor for stable estimation [16]. Given our sample size of 98, the initial plan to include 17 covariates yielded a subjects-to-predictor ratio of approximately 5.8 (98/17), falling below recommended thresholds. To mitigate overfitting, we adopted a hierarchical covariate adjustment strategy: baseline LVEF was retained as the core confounder, followed by sequential inclusion of demographic variables, cardiac biomarkers, and finally cardiovascular medications. This resulted in Model 4 with 8 predictors (N/p = 12.25), satisfying stability criteria. Collinearity diagnostics were assessed using the variance inflation factor (VIF), with VIF <5 indicating no significant multicollinearity. A two-sided *p*-value < 0.05 was considered statistically significant.

## 3. Results

### 3.1 Baseline Characteristics of the Study Cohort

Baseline characteristics between the control and SGLT2i groups were well-balanced, as shown in Table 2. The mean age of the study population was 63.21 ± 11.27 years, with 80.6% male and 76.5% having diabetes mellitus. Key baseline clinical parameters included mean blood glucose of 9.38 ± 3.21 mmol/L, HbA1c of 7.0% ± 1.12%, and eGFR of 90.48 ± 27.07 mL/min/1.73 m^2^. Vital signs were as follows: heart rate 80.92 ± 14.85 beats per minute, and blood pressure 134.81 ± 23.46/80.96 ± 14.54 mmHg. The mean low-density lipoprotein cholesterol (LDL-C) level was 3.19 ± 0.93 mmol/L. Other clinical factors, including history of hypertension, atrial fibrillation, and use of diuretics, beta-blockers, ARNI/ACEI/ARB, and lipid-lowering agents, were similarly distributed between the two groups (Table 2).

**Table 2.** **Baseline characteristics of the study population**.

		Control group (*n* = 49)	SGLT2i group (*n* = 49)	χ^2^/*t*/u	*p*-value
Age (years)		62.24 ± 11.28	64.18 ± 11.29	0.270	0.397
Male (no., %)		41 (83.7%)	38 (77.6%)	0.588	0.443
Smoking (no., %)		35 (71.4%)	32 (65.3%)	0.425	0.515
HR (bpm)		79.65 ± 14.24	82.18 ± 15.48	0.673	0.402
SBP (mmHg)		137.27 ± 26.18	132.24 ± 20.33	3.279	0.282
DBP (mmHg)		83.06 ± 13.95	78.86 ± 14.96	0.234	0.153
CK peak value (U/L)		2995.00 (781.50, 5087.00)	3124.00 (1100.00, 4620.50)	–0.206	0.837
NT-proBNP (pg/mL)		2172 (1451, 3921)	1940.50 (1166.75, 4469.5)	–0.725	0.496
HbA1c (%)		6.77 ± 0.83	7.19 ± 1.30	–1.889	0.063
Glucose (mmol/L)		9.22 ± 2.73	9.53 ± 3.65	–0.488	0.627
eGFR (mL/min/1.73 m^2^)		88.97 ± 30.18	91.98 ± 23.79	0.015	0.301
TC (mmol/L)		4.75 ± 1.12	4.75 ± 1.02	0.006	0.983
TG (mmol/L)		1.73 ± 1.58	1.65 ± 0.75	0.589	0.755
HDL-C (mmol/L)		1.11 ± 0.22	1.05 ± 0.21	0.071	0.227
LDL-C (mmol/L)		3.15 ± 0.95	3.23 ± 0.91	0.027	0.685
TC/HDL-C (%)		4.39 ± 1.10	4.63 ± 1.15	0.064	0.280
Comorbidities (no., %)					
	Hypertension	30 (61.2%)	32 (65.3%)	0.176	0.675
	Diabetes	40 (81.6%)	35 (71.4%)	1.420	0.233
	Stroke	9 (18.4%)	15 (30.6%)	1.986	0.159
	OMI	3 (6.1%)	4 (8.2%)	0.154	0.695
	Peripheral artery disease	26 (53.1%)	24 (49.0%)	0.163	0.686
	Dyslipidemia	20 (40.8%)	23 (46.9%)	0.373	0.541
	Atrial fibrillation	6 (12.2%)	6 (12.2%)	0.000	1.000
	Chronic kidney disease	6 (12.2%)	9 (18.4%)	0.708	0.400
Medications (no., %)					
	Diuretics	23 (46.9%)	26 (53.1%)	0.367	0.544
	MRA	27 (55.1%)	29 (59.2%)	0.167	0.683
	Beta–blockers	45 (91.8%)	44 (89.8%)	0.122	0.727
	ARB	12 (24.5%)	6 (12.2%)	2.450	0.118
	ARNI	36 (73.5%)	41 (83.7%)	1.515	0.218
	Antiplatelets	49 (100%)	49 (100%)	0.000	1.000
	Statin	48 (98.0%)	46 (93.9%)	1.043	0.307
	PCSK9i	2 (4.1%)	4 (8.2%)	0.710	0.399
Hypoglycemic medications (no., %)					
	Metformin	13 (26.5%)	12 (24.5%)	0.054	0.817
	Acarbose	8 (16.3%)	14 (28.6%)	2.110	0.146
	DPP4 inhibitors	8 (16.3%)	5 (10.2%)	0.798	0.372
	Others	17 (34.7%)	19 (38.8%)	0.176	0.675
Killip class (no., %)				2.262	0.520
	I	26 (53.1%)	27 (55.1%)		
	II	20 (40.8%)	17 (34.7%)		
	III	0 (0%)	2 (4.1%)		
	IV	3 (6.1%)	3 (6.1%)		

Notes: Data are presented as mean ± SD, n (%), or median (quartile). Normally distributed variables were used for independent samples *t*-tests or paired samples *t*-tests, abnormally distributed variables were used for Mann–Whitney U test. χ^2^ or Fisher test for categorical variables. *p* < 0.05 was considered statistically significant. Abbreviations: HR, heart rate; SBP, systolic blood pressure; DBP, diastolic blood pressure; CK peak value, creatine kinase peak value; NT-proBNP, N-terminal pro-B-type natriuretic peptide; HbA1c, glycated hemoglobin; eGFR, estimated glomerular filtration rate; TC, total cholesterol; TG, triglyceride; HDL-C, high-density lipoprotein cholesterol; LDL-C, low-density lipoprotein cholesterol; TC/HDL-C, total cholesterol/high-density lipoprotein cholesterol; OMI, old myocardial infarction; MRA, mineralocorticoid receptor antagonist; ARB, angiotensin receptor blocker; ARNI, angiotensin receptor-neprilysin inhibitor; PCSK9i, proprotein convertase subtilisin/kexin type 9 inhibitor; DPP4 inhibitors, dipeptidyl peptidase 4 (DPP-4) inhibitors.

### 3.2 Effect of SGLT2i on LVEF

**Fig. 2.**
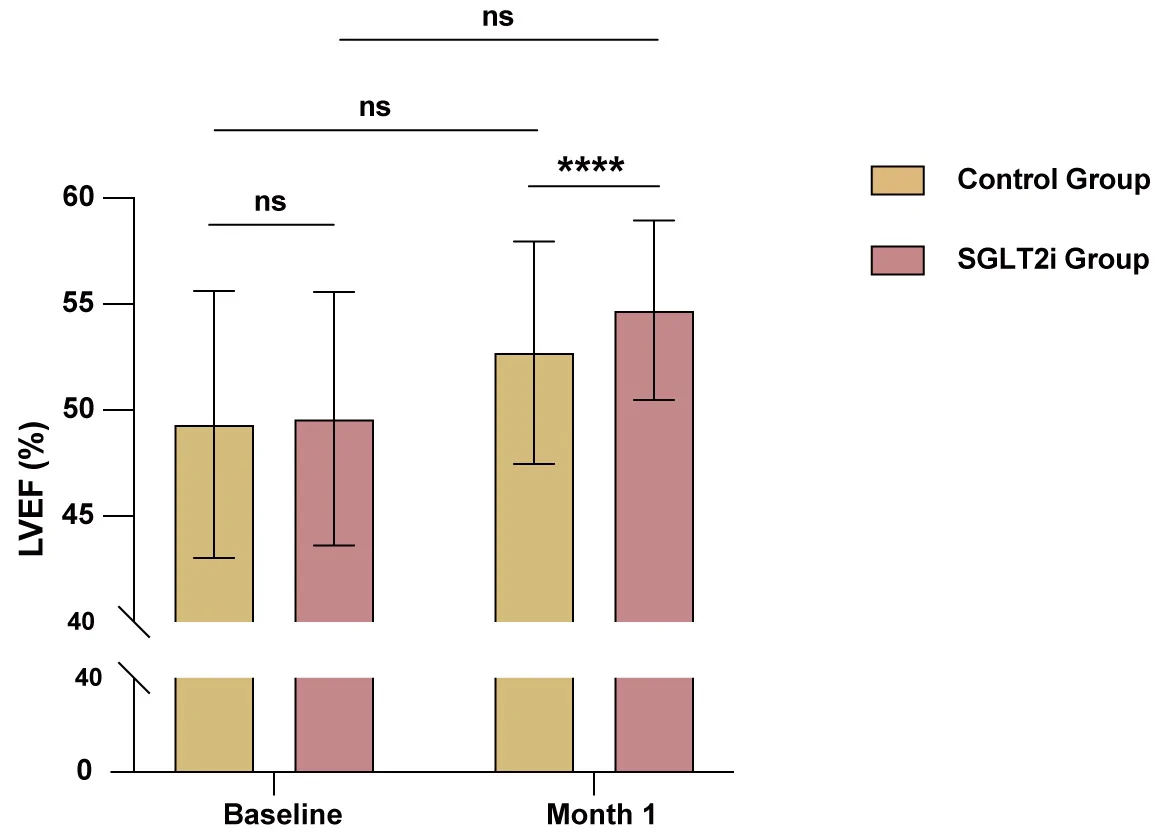
**Comparison of LVEF between groups at baseline and at one-month follow-up**. Notes: Data are presented as mean ± SD. Within-group comparisons were performed using paired samples *t*-tests and between-group differences were analyzed using independent samples *t*-tests. ns: *p* > 0.05, ****p < 0.0001. LVEF, left ventricular ejection fraction.

As shown in Fig. 2, baseline LVEF was comparable between the SGLT2i group (49.59% ± 5.98%) and the control group (49.33% ± 6.29%), with no statistically significant difference (*p* > 0.05). Following treatment, LVEF in both the control group (52.71% ± 5.25%) and the SGLT2i group (54.71% ± 4.24%) showed a significant increase from baseline (both *p* < 0.05). As shown in Table 3, in the unadjusted independent samples *t*-test (Model 1), early SGLT2i initiation was associated with a 1.74% absolute increase in LVEF compared to controls (95% CI: –0.46, 3.93), though this difference did not reach statistical significance (*p* = 0.119). After adjusting for baseline LVEF using analysis of covariance (ANCOVA, Model 2), early SGLT2i initiation was independently associated with significant LVEF improvement at one month, with an effect size of 1.89% (95% CI: 0.25, 3.53, *p* = 0.024). In multivariable linear regression models, Model 3 (adjusted for baseline LVEF, age, gender, and CK peak) demonstrated a 2.00% absolute improvement in LVEF (95% CI: 0.47, 3.54, *p* = 0.011); with further adjustment for concomitant medications (Model 4), the effect estimate was 1.92% (95% CI: 0.44, 3.40, *p* = 0.012).

**Table 3.** **Effect of early SGLT2i initiation on LVEF improvement at one month**.

Model	Effect size (%, 95% CI)	*p* value	Adjusted R^2^
Model 1	1.74 (–0.46, 3.93)	0.119	-
Model 2	1.89 (0.25, 3.53)	0.024 *	0.290
Model 3	2.00 (0.47, 3.54)	0.011 *	0.388
Model 4	1.92 (0.44, 3.40)	0.012 *	0.433

Model 1: Independent samples *t*-test (unadjusted). Model 2: ANCOVA with baseline LVEF as covariate (group variable as fixed factor). Model 3: Multivariable linear regression adjusted for group variable, baseline LVEF, age, gender, and CK peak. Model 4: Multivariable linear regression adjusted for group variable, baseline LVEF, age, gender, CK peak, MRA, beta-blockers, and ARB/ARNI. *p < 0.05. CI, confidence intervals; ANCOVA, analysis of covariance.

From Model 2 to Model 4, effect estimates remained stable (1.89%–2.00%) with overlapping 95% confidence intervals and consistent statistical significance (all *p* < 0.05). Collinearity diagnostics revealed no significant multicollinearity, with all VIF <1.8. The progressive increase in adjusted R^2^ (0.290→0.388→0.433) indicates improved model fit without overfitting. The results support a potential benefit of early initiation of the SGLT2i on LVEF.

### 3.3 Changes in NT-proBNP Level

**Fig. 3.**
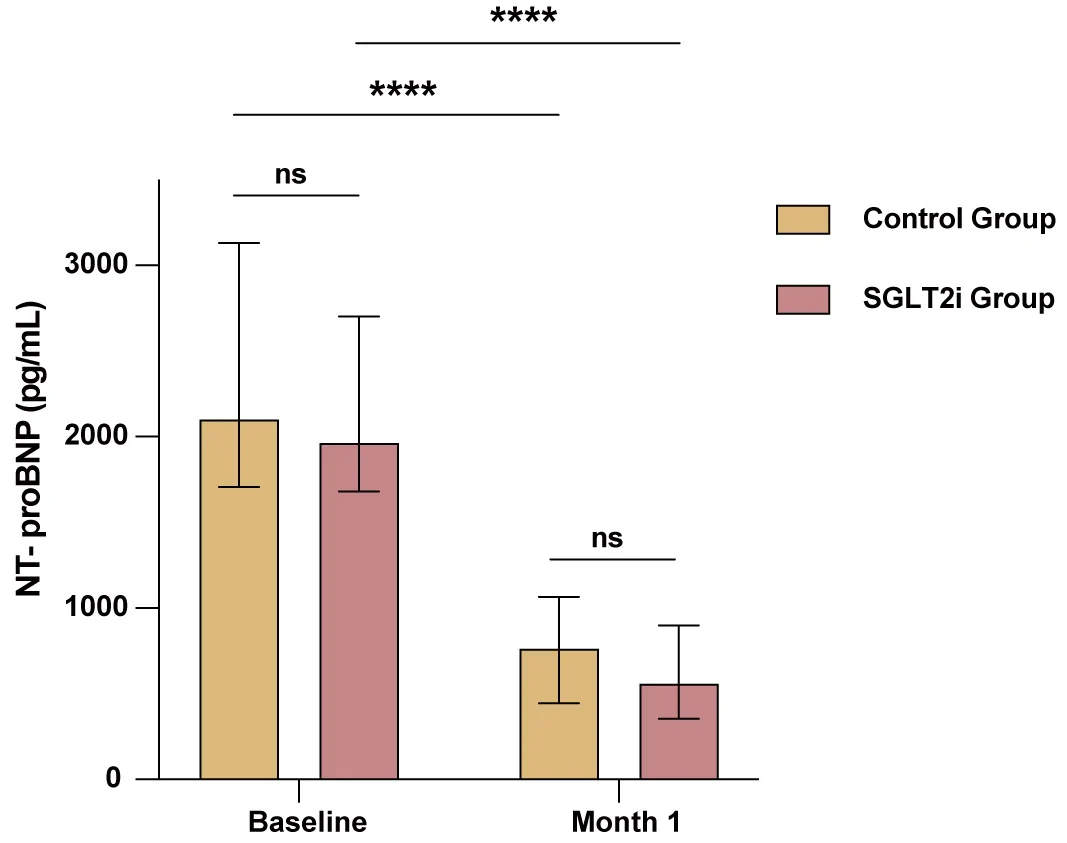
**Comparison of NT-proBNP level between groups at baseline and at one-month follow-up**. Notes: Data are presented as median (quartiles). Within-group comparisons were performed using the Wilcoxon signed-rank test and between-group differences were analyzed using the Mann-Whitney U test. ns: *p* > 0.05, ****p < 0.0001. NT-proBNP, N-terminal pro-B-type natriuretic peptide.

As shown in Fig. 3, baseline NT-proBNP levels were comparable between the SGLT2i group [1940.50 (IQR: 1166.75, 4469.5) pg/mL] and the control group [2172 (IQR: 1451, 3921) pg/mL], with no statistically significant difference (*p* > 0.05). Following treatment, within-group analysis revealed that NT-proBNP levels decreased significantly from baseline in both the control group [581.90 (IQR: 351.55, 1503.50) pg/mL] and the SGLT2i group [713.60 (IQR: 255.07, 1358.50) pg/mL] (both *p* < 0.05). However, no significant difference was observed between the two groups at one-month follow-up (*p* > 0.05).

### 3.4 Comparison of Renal Function Parameters

As shown in Table 4, baseline renal function parameters were comparable between the two groups, with no statistically significant differences observed (all *p* > 0.05). Within-group analysis revealed there were no significant changes in uric acid levels from baseline to follow-up in either group (*p* > 0.05). However, blood urea nitrogen (BUN) and creatinine (Cr) levels increased significantly from baseline in both groups (*p* < 0.05), while the eGFR decreased significantly in both groups (*p* < 0.05).

**Table 4.** **Comparison of renal function parameters between groups (X̅ ± SD)**.

		Control group (*n* = 49)	SGLT2i group (*n* = 49)	*t*	*p*-value
BUN (mmol/L)	Baseline	5.21* ± *1.82	5.26* ± *1.73	0.220	0.874
	One month	5.89* ± *1.70 ^#^	6.49* ± *1.99 ^#^	0.199	0.117
Cr (µmol/L)	Baseline	77.20* ± *22.26	69.12* ± *18.36	0.609	0.053
	One month	86.69* ± *23.43 ^#^	77.83* ± *23.00 ^#^	0.544	0.062
UA (µmol/L)	Baseline	350.10* ± *100.39	320.67* ± *99.04	0.031	0.147
	One month	365.76* ± *117.46	306.51* ± *87.62	2.144	0.006 *
eGFR (mL/min/1.73 m^2^)	Baseline	88.97* ± *30.18	91.98* ± *23.79	0.739	0.585
	One month	80.60* ± *30.00 ^#^	82.41* ± *23.23 ^#^	0.333	0.740

Notes: Data are presented as mean ± SD. Within-group comparisons were performed using paired *t*-tests. Between-group comparisons at each time point were performed using independent *t*-tests. *p* < 0.05 was considered statistically significant. ^#^ *p* < 0.05 vs. baseline within the same group; *p < 0.05 vs. control group at the same time point. Abbreviations: BUN, blood urea nitrogen; Cr, creatinine; UA, uric acid; eGFR, estimated glomerular filtration rate.

At one-month follow-up, between-group comparison revealed the uric acid level was significantly lower in the SGLT2i group (306.51 ± 87.62 μmol/L) compared with the control group (365.76 ± 117.46 μmol/L) (*p* < 0.05). However, no significant differences were observed between the two groups for BUN, Cr, or eGFR (all *p* > 0.05).

### 3.5 Comparison of Liver Function, Lipid Profile, and Potassium Level

The serum potassium level did not differ significantly between the two groups at baseline or at one-month follow-up (Table 5). However, within-group analysis revealed a significant increase in the serum potassium level from baseline in the control group (*p* < 0.05), but not in the SGLT2i group. Regarding liver function, the alanine aminotransferase (ALT) level decreased significantly from baseline in the SGLT2i group (*p* < 0.05), but not in the control group. Between-group analysis revealed no significant differences in gamma-glutamyl transferase (GGT), aspartate aminotransferase (AST), total cholesterol (TC), triglycerides (TG), high-density lipoprotein cholesterol (HDL-C), low-density lipoprotein cholesterol (LDL-C), or the TC/HDL-C ratio at either baseline or one-month follow-up.

**Table 5.** **Comparison of potassium, liver function, and lipid profiles (X̅ ± SD)**.

		Control group (*n* = 49)	SGLT2i group (*n* = 49)	*t*	*p* value
K^+^ (mmol/L)	Baseline	3.92 ± 0.33	3.98 ± 0.39	0.343	0.451
	One month	4.16 ± 0.50 ^#^	4.08 ± 0.37	0.365	0.390
GGT (U/L)	Baseline	29.62 ± 29.86	33.60 ± 31.88	0.403	0.524
	One month	33.68 ± 22.03	34.65 ± 25.97	0.543	0.843
ALT (U/L)	Baseline	50.21 ± 27.25	54.85 ± 34.67	1.605	0.463
	One month	33.67 ± 53.06	28.22 ± 30.19 ^#^	0.985	0.533
AST (U/L)	Baseline	234.44 ± 182.43	251.94 ± 188.17	0.002	0.641
	One month	22.62 ± 7.90 ^#^	22.40 ± 4.84 ^#^	8.483	0.873
TC (mmol/L)	Baseline	4.75 ± 1.12	4.75 ± 1.02	0.006	0.983
	One month	3.09 ± 1.17^ #^	3.21 ± 1.05 ^#^	0.141	0.598
TG (mmol/L)	Baseline	1.73 ± 1.58	1.65 ± 0.75	0.589	0.755
	One month	1.51 ± 0.59	1.48 ± 0.77	1.690	0.879
HDL-C (mmol/L)	Baseline	1.11 ± 0.22	1.05 ± 0.21	0.071	0.227
	One month	1.13 ± 0.46	1.07 ± 0.21	1.686	0.352
LDL-C (mmol/L)	Baseline	3.15 ± 0.95	3.23 ± 0.91	0.027	0.685
	One month	1.88 ± 0.77 ^#^	1.93 ± 0.80 ^#^	0.496	0.765
TC/HDL-C	Baseline	4.39 ± 1.10	4.63 ± 1.15	0.064	0.280
	One month	2.91 ± 1.10 ^#^	3.07 ± 1.03 ^#^	0.114	0.453

Notes: Data are presented as mean ± SD. Within-group comparisons were performed using paired *t*-tests. Between-group comparisons at each time point were performed using independent *t*-tests. *p* < 0.05 was considered statistically significant. ^#^ *p* < 0.05 vs. baseline within the same group. Abbreviations: GGT, gamma-glutamyl transferase; ALT, alanine aminotransferase; AST, aspartate aminotransferase; TC, total cholesterol; TG, triglycerides; HDL-C, high-density lipoprotein cholesterol; LDL-C, low-density lipoprotein cholesterol; TC/HDL-c, total cholesterol/high-density lipoprotein cholesterol.

### 3.6 Adverse Events and Readmission for Heart Failure

The incidence of adverse events and readmission for HF is summarized in Table 6. Hypotension occurred in two patients in the control group and three patients in the SGLT2i group. Hyperkalemia was reported in one patient in the control group, while no cases were observed in the SGLT2i group. No severe renal deterioration or ketoacidosis events were observed in either group. A urinary tract infection was reported in one patient in the SGLT2i group. Readmission for HF occurred in three patients in the control group and two in the SGLT2i group. Overall, no statistically significant differences were observed between the two groups in terms of the incidence of adverse events or readmission for HF (both *p* > 0.05).

**Table 6.** **Safety and adverse events**.

	Control group (*n* = 49)	SGLT2i group (*n* = 49)
Hypotension	2	3
Hyperkalemia	1	0
Severe deterioration of renal function	0	0
Urinary tract infection	0	1
Ketoacidosis	0	0
Hospitalization for HF	3	2

Abbreviations: HF, heart failure.

## 4. Discussion

In this study of 98 patients with acute anterior STEMI and heart failure, early empagliflozin initiation was associated with higher LVEF at one-month follow-up (adjusted effect size: 1.92%, *p* = 0.012). The SGLT2i group also showed a trend toward reduced uric acid levels, with no significant differences in safety outcomes between groups. However, this effect size lies within the reported measurement variability (2.8% ± 1.5%), suggesting that this change may be partially attributable to measurement error. Consequently, this LVEF improvement cannot be interpreted as evidence of meaningful ventricular remodeling, and its clinical significance remains uncertain. Large-scale prospective studies are needed to validate its impact on long-term hard endpoints.

The improvement in LVEF observed in our study is consistent with the findings of recent meta-analyses. A meta-analysis comprising 7 randomized controlled trials (11,407 patients with acute MI) reported that while SGLT2i did not significantly reduce major adverse cardiovascular events or all-cause mortality, they were associated with a 24% reduction in HF risk (risk ratio [RR] = 0.76, 95% CI: 0.63–0.91) and a significant improvement in LVEF (mean difference [MD] = 1.86%, 95% CI: 1.58–2.14) [17]. A retrospective study of 100 patients with type 2 diabetes and AMI found that dapagliflozin significantly improved NT-proBNP levels and cardiac function parameters (left ventricular end-diastolic and end-systolic diameters) at 6 months compared with controls [18]. The EMMY randomized controlled trial further corroborated these findings by demonstrating that empagliflozin treatment for 26 weeks significantly reduced NT-proBNP levels, as well as improving LVEF (1.5%, 95% CI: 0.2–2.9%) and cardiac structural parameters [19]. Together with the current findings, these results suggest a benefit from early intervention with SGLT2i on cardiac remodeling. However, despite evidence from meta-analyses, real-world studies, and the EMMY trial indicated potential benefits in the acute ischemic setting [20], the recent DAPA-MI and EMPACT-MI trials both reported neutral primary endpoints.

Although our findings may at first appear to differ from the neutral primary endpoint results of the two large randomized controlled trials, DAPA-MI and EMPACT-MI, a deeper analysis reconciles these observations. In the DAPA-MI trial, the primary hierarchical composite outcome significantly favored dapagliflozin over placebo using a win-ratio approach (win ratio 1.34, 95% CI: 1.20–1.50), with the benefit largely attributed to improvements in cardiometabolic outcomes [9]. Although EMPACT-MI did not meet its primary endpoint (all-cause death or HF hospitalization; HR = 0.90, 95% CI: 0.76–1.06), secondary analyses revealed that empagliflozin significantly reduced the risk of first HF hospitalization (HR = 0.77, 95% CI: 0.60–0.98) and total HF hospitalizations (rate ratio 0.67, 95% CI: 0.51–0.89) [21]. Hence, both large trials confirmed that SGLT2 inhibitors confer HF and metabolic benefits post-MI, although these did not translate into early reduction of mortality. The differences between our study and these trials should be viewed through several lenses. First, with regard to population selection, our study focused on a high-risk subgroup with established HF following anterior MI. The risk of adverse remodeling in this subgroup is 1.9 times higher than that of non-anterior MI [11], which theoretically offers a greater potential for benefit. Second, with regard to endpoint selection, LVEF as a sensitive surrogate endpoint can capture early effects in a smaller sample, whereas hard endpoints require larger sample sizes and longer follow-up. Third, regarding the observation time point, a one-month follow-up may capture early hemodynamic effects of SGLT2i (e.g., diuresis reducing volume load), whereas improvements in long-term remodeling and clinical hard endpoints may require more prolonged treatment to become apparent. Although this study evaluated only clinical endpoints and was not designed to investigate molecular mechanisms, hypothetical mechanisms proposed in prior preclinical studies include ion homeostasis regulation and cell energy metabolism-related pathways, which may be associated with the observed LVEF changes [22,23,24] and require further validation in basic experimental studies.

Current ESC and ACC/AHA guidelines for HF recommend SGLT2i as core therapy for heart failure with mildly reduced ejection fraction (HFmrEF) and heart failure with preserved ejection fraction (HFpEF) (Class I, Level A) [25]. However, their routine use is not yet explicitly recommended for patients with AMI, irrespective of whether they have concomitant HF or a history of HF. Our study, along with other remodeling studies, suggests that early initiation of SGLT2i in high-risk patients with HF after anterior MI can provide short-term improvements in cardiac function, offering new clinical evidence for this subgroup not currently covered by the guidelines. However, since LVEF is a surrogate endpoint, the observed improvements require validation against hard clinical outcomes in larger prospective studies.

Exploratory analyses showed that changes in the NT-proBNP levels were indicators of symptomatic HF improvement in both groups. However, SGLT2i did not confer an additional natriuretic peptide-lowering effect in the short-term. No significant difference in renal function between groups was observed (Table 4). The significant reduction in serum uric acid level observed with SGLT2i in the present study aligns with previous reports in diabetic and HF populations [26,27]. Hyperuricemia is not only a major cause of gout, but also an independent risk factor for MI [28]. A meta-analysis reported that SGLT2i reduce uric acid by an average of 37.73 μmol/L, with empagliflozin showing the most potent effect [29]. Mechanistically, SGLT2i may lower uric acid by interfering with urate reabsorption mediated by glucose transporter 9 (GLUT9) in the renal tubules [30]. However, as an exploratory endpoint, the clinical significance of changes in uric acid requires further investigation [31].

## 5. Limitations

This study has several limitations: (1) The single-center retrospective design is subject to inherent biases; (2) The relatively small sample size limits the power for subgroup analyses; (3) The follow-up period of only one month precludes assessment of long-term outcomes; (4) The small number of non-diabetic patients (n = 23) means the conclusions cannot be extrapolated to this population; (5) As a surrogate endpoint, the association between LVEF improvement and long-term hard outcomes requires further validation; (6) The initial design including 17 covariates raised concerns for overfitting. Therefore, we report only the parsimonious model comprising the group variable and seven core covariates.

## 6. Conclusion

In hemodynamically stable patients with acute anterior STEMI complicated by heart failure, we observed that early SGLT2i initiation was associated with higher LVEF at one month, with a trend toward reduced uric acid levels. No significant differences in safety outcomes were observed between groups. Given the study design limitations and the effect size falling within measurement variability, these findings should be considered hypothesis-generating and cannot serve as a basis for clinical decision-making, and large-scale prospective studies are needed to validate their impact on long-term clinical outcomes.

## Data Availability

The data supporting the findings of this study are available from the corresponding author upon reasonable request.
